# Getting into the water: a prospective observational study of water immersion for labour and birth at a New Zealand District Health Board

**DOI:** 10.1186/s12884-020-03007-6

**Published:** 2020-05-20

**Authors:** Robyn M. Maude, Mikyung Kim

**Affiliations:** School of Nursing, Midwifery, and Health Practice, Faculty of Health, Victoria University of Wellington, PO Box 7625, Newtown, Wellington, 6242 New Zealand

## Background

Water immersion during labour and birth offers birthing women a means of non-pharmacological pain relief and a reduction in unnecessary and often routine intrapartum interventions [1]. Women who labour and birth in water are more likely to birth spontaneously and physiologically and experience fewer intrapartum interventions [2–4]. For the neonates of women who use water immersion during labour and birth, there are no additional risks compared to neonates born to women who do not use water immersion during labour and birth [5, 6].

In New Zealand, the use of water immersion during labour and birth is predominantly at home or in primary maternity facilities (midwife-led birthing units) for low-risk women. Some secondary maternity units (for both uncomplicated and complicated pregnancies and births supported by multidisciplinary teams and access to operating theatres), and tertiary maternity units (for women with high-risk, complicated pregnancies, specialist services and level 3 neonatal intensive care unit) provide access to birth pools or baths for ‘low risk’ women to utilise during labour and birth [4]. For women without access to a primary birthing facility, the secondary or tertiary maternity unit may be her only option to use water immersion for labour and birth.

The New Zealand maternity system is an integrated system of primary, secondary and tertiary care that is free for most women. A Lead Maternity Carer (LMC) of the woman’s choice is responsible for care throughout pregnancy, labour and birth, and postnatally to 6 weeks. The LMC can be a midwife, a general practitioner (family physician) with a diploma in obstetrics or a private obstetrician (who can charge a fee for service). The majority (93.6%) of pregnant women in New Zealand choose a case-loading community-based LMC midwife who provides continuity-of-care in the woman’s home, a midwife-led unit, or a secondary or tertiary hospital [7]. If the maternity care becomes complicated, the case-loading community-based LMC midwife will refer the woman to the obstetric specialist team at a secondary or tertiary hospital. The obstetric specialist team will work with the hospital-employed midwives (also known as core midwives) (50.7% of the midwifery workforce) [8], either to support the case-loading community-based LMC midwife to continue to provide care or to provide the woman’s care following transfer of care.

There is a dearth of data available from secondary and tertiary maternity units where water immersion for labour and birth are offered, particularly in New Zealand. The lack of data is likely to be due to a lack of capacity of the information technology (IT) systems to record water immersion and water births. No complete national data is reporting on water immersion and water births across all maternity settings [7]. The only formal reporting of the use of water immersion for labour and birth comes from the Midwifery and Maternity Provider Organisation Ltd. (MMPO) from their midwife members [8]. In 2016, the most recently available published data, the percentage of water births was 10.8%, although 26.8% of women in the report sample used water immersion during labour. Women who gave birth at a primary facility had a higher proportion of water births (34.8%) than those birthing at secondary or tertiary facilities (7 and 2.9% respectively) [8]. There is a need to highlight the practice of and outcomes of water immersion and water birth in the New Zealand context. The research question guiding this study was: What are the characteristics and outcomes for women and their babies who used water immersion for labour and birth in maternity facilities in one New Zealand District Health Board?

## Methods

This study aimed to describe the maternal characteristics, intrapartum events, interventions, and maternal and neonatal outcomes of women who used water immersion during labour and birth at one New Zealand District Health Board (DHB). This paper presents the results from a prospective observational study of women who used water immersion for labour and birth across the three maternity facilities in the DHB from February 2009 to March 2014.

### Setting

The DHB maternity services include a tertiary-level maternity unit in a New Zealand city (average 3800 births per year) plus two midwife-led units (MLUs) (one at 30 mins and the other at 60 min away from the tertiary referral unit). At the tertiary maternity unit, there are 12 birth rooms each with the same design features, including a birth pool or bath, included in the new hospital design commissioned in 2009. The room size and layout differ slightly. Five rooms have a purpose-built birth pool while the remaining seven rooms have baths permanently installed in the corner of the room. The birth pools are round, 1200 mm in diameter and 650 mm deep. There is access around 66% of the pool, a shower, two handrails and steps into the pool. The baths are five-sided and are bound on two sides by a wall. The sides alongside the wall measure 1480 mm, the two short slides measure 670 mm and the front access side measures 1080 mm. The depth of the bathtub is 510 mm. There is a hand-held mixer shower hose set plumbed in over the bath, four handrails, a sieve and thermometer and easy access to piped oxygen, suction and Entonox. At MLU 1 there is one bath measuring 1000 mm wide × 1840 mm long and 630 mm deep. The bath is in a separate room attached to two birth rooms. There is access around three sides of the bath, inner armrests and a headrest. All pools use a disposable liner. For comfort, a soft mat under the liner, and a floor mat are available for comfort for kneeling/laying on the floor around the bath. Equipment in the bathroom includes: a sieve, thermometer, a large mirror and an electric oil burner, portable oxygen, suction and Entonox are available. At MLU 2 there is one bathroom with a purpose-built birth pool permanently installed towards the corner of the room adjacent to the only birth room. The birth pool is rectangular, measuring 1020 mm wide, 1350 mm long and 740 mm deep. There is a built-in seat which can also be used as a step to get in and out, with access around three sides of the pool.

### Participants

Women who used the birth pool or bath during labour and birth across all three DHB maternity facilities, initiated at the time the new tertiary unit opened in February 2009 through to March 2014. Criteria for the use of water immersion for labour and birth usage, as outlined in the DHB guideline, as at least 37 weeks gestation, no adverse fetal or maternal factors in the pregnancy, an informed choice, established labour (judicious use for women with long latent labour is useful to promote relaxation) when there has been a diagnosis of labour dystocia (before using oxytocin). Ruptured membranes are not a contraindication for the use of the birth pool or bath.

### Data collection

A paper-based data collection tool, included in the medical record of all women booked to birth at each of the three facilities, was adapted from the DHB water immersion guideline and the literature on water immersion. The form captured details of place of birth, type of caregiver and type of water facility as well as maternal characteristics (such as parity and gestation); and intrapartum events and interventions (such as labour onset, membrane rupture, vaginal examinations, pain relief). Maternal outcomes (such as length of labour, mode of birth, estimated blood loss, third stage technique, perineal or vaginal wall tear, labial tear, episiotomy, suturing) and neonatal outcomes (Apgar scores and NICU admission) were included (see supplementary file 1).

Lead Maternity Care (LMC) midwives or core (hospital employed) midwives were asked to complete the form for each woman who used water immersion during labour or birth. Midwives could also enter the presence of any risk factors from the current or previous pregnancies. Data were manually entered in Excel spreadsheet by the primary investigator and research assistant and transferred to SPSS Version 23 for analysis. Where data was unclear or missing, a review of other DHB data sources such as the DHB Patient Information Management System (PIMS) and clinical records of women and neonates (if admitted to Neonatal Intensive Care Unit (NICU) post-birth) occurred. The denominator of some variables differs given missing data.

### Data analysis

Descriptive statistics describe the characteristics of women who used water immersion during labour and birth and to describe the women and infants’ birthing outcomes. Frequencies and percentages were calculated for categorical variables, mean and standard deviation for continuous variables.

### Ethics approval

The New Zealand Health and Disability Ethics Committee (Approval number 15/CEN/55) provided research ethics approval.

## Results

There were 1517 data collection forms completed for women who used a birth-pool or bath during labour and birth between February 2009 and March 2014: 1188 from the tertiary maternity unit and 329 from two midwifery-led units (164 from MLU 1 and 165 from MLU 2). There were approximately 19,628 births at the DHB facilities during the study period, indicating approximately 7.7% of women used water immersion for labour and birth. As the data collection was reliant on midwives manually completing the data collection form, we cannot be sure this number (1517) fully represented all women who used water for labour and birth during the data collection period.

Of the women for whom a data form was completed (1517) there were 584 (38.5%) who had a water birth, 1275 (84%) had a spontaneous vaginal birth, 242 (16%) had assisted births or caesarean sections. Midwives attending women during labour and birth were community-based LMCs (92.7%), core midwife/team (5.0%) and medical LMC/core midwife (2.3%).

**Fig. 1:**
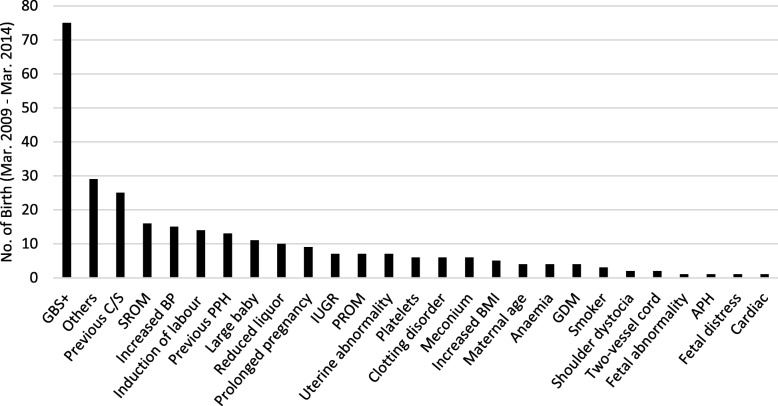
Risk factors in the current or any previous pregnancy or birth. APH = Antepartum Haemorrhage; BMI = Body Mass Index; BP = Blood Pressure; C/S = Caesarean Section; GBS = Group B Streptococcus; GDM = Gestational Diabetes Mellitus; IUGR = Intrauterine Growth Restriction; PPH = Postpartum Haemorrhage; PROM = Premature Rupture of Membranes; SROM = Spontaneous Rupture of Membranes

There were 284 (18.7%) data sheets that recorded risk factors for women who used water immersion for labour and birth during the data collection period. Examples of the risk factors recorded are in Fig. 1. The risk factors reported as ‘others’ (10.2%) included oxytocin infusion, hydramnios, deflexed occipito-posterior position (OP), small for dates baby (SFD), maternal mental health, and haemophilia.

A little over half of the women in this study were nulliparous (*n* = 830, 54.7%). Most women had a gestational age at labour onset of more than 38 weeks up to 42 weeks gestation (94.3%). There were four women with a gestation of fewer than 36 weeks (0.3%) and ten women whose gestation was more than 42 weeks (0.7%) included in the analysis. Most (93.8%) women’s labour began spontaneously. The woman whose gestational age was less than 36 weeks and the three women whose gestational age were more than 42 weeks gestation had a water birth without any maternal and neonatal complications.

Nearly 56% of women used a birth pool and 44.3% used a bath. The mean cervical dilatation on entry to water was 5.6 cm (SD 2.1), and the mean number of contractions on entry to water in 10 min was 3.4 (SD 0.7). Water temperature on entry to a birth pool or bath ranged from 23 °C to 44 °C (mean 37.4 °C, SD1.3), and the exit temperature was between 24 °C and 42 °C (mean 36.7 °C, SD 1.32).

**Fig. 2:**
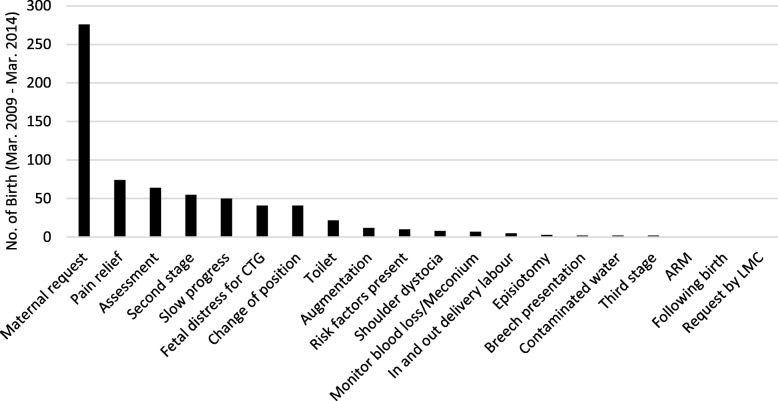
Reasons for leaving the birth pool/bath before birth. ARM = Artificial Membrane Rupture; CTG = Cardiotocography; LMC = Lead Maternity Care

Of the total 1517 women who used a birth pool or bath during labour, 677 women (44.6%) left the birth pool or bath before birth. Just over 40% of women left the water at their request as they felt fatigued/unwell/hot/cold. Others left due to a need for pharmacological pain relief (10.9%), assessment (9.5%), second stage (8.1%), slow progress (7.4%), fetal distress for cardiotocography (CTG, 6.1%), change of position (6.1%), and to use the toilet (3.2%) (Fig. 2).

### Effects of water immersion for labour and birth on intrapartum events, interventions and maternal and neonatal outcomes

From the 1517 women who used water immersion for labour and birth there were 584 (38.5%) water births. More than half (53.7%) of the water births occurred in multiparous women whereas only a quarter (25.9%) of nulliparous had a water birth. Overall, the rate of spontaneous vaginal birth was 84% with 9.4% assisted deliveries and 6.5% caesarean sections. The proportion of assisted birth/CS was significantly higher in nulliparous (25.7%) than in multiparous (4.2%) (*p* = < 0.001) (Table 1).

**Table 1:** Characteristics and outcomes for omen and babies who used water immersion for labour and birth

Characteristics and outcomes		
	Frequency	Percent
**Caregiver in labour**		
Self -employed Midwife	1406	92.7
Core Midwife	76	5.0
Dr. LMC/Core Midwife	35	2.3
Total	1517	100.0
**Parity**		
Nulliparous	830	54.7
Multiparous	687	45.3
Total	1517	100.0
**Place of Birth**		
Tertiary Unit	1188	78.3
Midwife-led Unit 1	164	10.8
Midwife-led Unit 2	165	10.9
Total	1517	100.0
**Gestation at start of labour**		
< 36 weeks	4	0.3
36–37^6^ weeks	72	4.7
38–39^6^ weeks	649	42.8
40–41^6^ weeks	782	51.5
> 42 weeks	10	0.7
Total	1517	100.0
**Onset of Labour**		
Spontaneous	1423	93.8
Induced	93	6.1
Missing	1	0.1
Total	1517	100.0
**Membrane Rupture**		
Spontaneous	1128	74.4
Artificial	380	25.0
Intact	5	0.3
Missing	4	0.3
Total	1517	100.0
**Cervical dilatation on entry to water (cm)**		
0	2	0.1
1	17	1.1
2	47	3.1
3	126	8.3
4	215	14.2
5	251	16.5
6	167	11.0
7	135	8.9
8	130	8.6
9	61	4.0
10	69	4.5
Missing	297	19.6
Total	1517	100.0
Mean = 5.6cms (SD 2.1)		
**No. of contractions on entry to water in 10mins**		
1	3	0.2
2	83	5.5
3	678	44.7
4	511	33.7
5	44	2.9
6	4	0.3
Missing	194	12.8
Total	1517	100.0
Mean = 3.4 (SD 0.7)		
**Water Temperature on entry to Pool (°C)**		
Below 35 degrees	18	1.2
35–35.9	14	0.9
36–36.9	222	14.6
37–37.9	482	31.8
38–38.9	346	22.8
39–39.9	117	7.7
Over 40 degrees	41	2.7
Missing	277	18.3
Total	1517	100.0
Mean = 37.4 (SD 1.3)		
**Pain Relief before entry to water**		
Yes	584	38.5
No	933	61.5
Total	1517	100.0
**Pain Relief after leaving the pool**		
Yes	555	36.6
No	962	63.4
Total	1517	100.0
**Number of Vaginal Examinations**		
None	1160	76.5
1	280	18.5
2	65	4.3
3+	9	0.6
Missing	3	0.2
Total	1517	100.0
**Length of 1st stage of labour (mins)**		
Mean = 330 (SD 238)		
**Length of 2nd stage of labour (mins)**		
Mean = 55 (SD 62)		
**Length of 3rd stage of labour (mins)**		
Mean = 16 (SD 17)		
**Mode of Birth**		
SVD OA	1250	82.4
SVD OP	25	1.6
Assisted delivery	143	9.4
CS	99	6.5
Total	1517	100.0
**Birth underwater**		
Yes	584	38.5
No	933	61.5
Total	1517	100.0
**Position of mother at time of birth**		
Sitting	25	1.6
Kneeling	133	8.8
Standing	39	2.6
Hands and Knees	239	15.8
Lateral	34	2.2
Semi-reclined	632	41.7
Lithotomy	182	12.0
Squatting	83	5.5
Lying flat	122	8.0
Floating	2	0.1
Others	2	0.1
Missing	24	1.6
Total	1517	100.0
**Third stage management**		
Active	917	60.4
Physiological	594	39.2
Both	6	0.4
Total	1517	100.0
**Estimated blood loss**		
0–499	1322	87.1
500–999	141	9.3
1000–1499	26	1.7
> 1500	13	0.9
Missing	15	1.0
Total	1517	100.0
**Perineal and/or vaginal wall tear**		
No	648	42.7
Yes 1st degree	390	25.7
Yes 2nd degree	415	27.4
Yes 3rd degree	33	2.2
Yes 4th degree	2	0.1
Yes Graze	20	1.3
Missing	9	0.6
Total	1517	100.0
**Episiotomy**		
Yes	213	14.0
No	1294	85.3
Missing	10	0.7
Total	1517	100.0
**Suturing of Tear/Episiotomy**		
Yes	720	47.5
No	784	51.7
Missing	13	0.9
Total	1517	100.0
**Apgar score at 1 min**		
< 7	90	5.9
≥ 7	1421	93.7
Missing	6	0.4
Total	1517	100.0
**Apgar score at 5 mins**		
> 7	11	0.7
≥ 7	1499	98.8
Missing	7	0.5
Total	1517	100.0
**Apgar score at 10 mins**		
< 7	4	0.3
≥ 7	1038	68.4
Missing	475	31.3
Total	1517	100.0
**Admission to NICU**		
Yes	37	2.4
No	1474	97.2
Missing	6	0.4
Total	1517	100.0

A quarter of the women had an artificial rupture of membranes (ARM). More than a third (*n* = 555, 36.6%) of women in this study used pain relief after leaving the pool or bath (Table 1). The pharmacological pain relief commonly used were Inhalation/Entonox, Epidural, and Opioid. Alternative and complementary therapies used were transcutaneous nerve stimulation (TENS), acupuncture, acupressure, homeopathy, massage, and heat-pack.

The mean duration of the first stage of labour was 330 mins (SD 238), the second stage of labour was 55 mins (SD 62) and the third stage of labour was 16 mins (SD 17) (Table 1).

Women generally preferred to adopt a semi-reclined (41.7%) or a hands and knees position (15.8%) for birth. Placental birth was actively managed in 917 (60.4%) women whilst 594 (39.2%) has a physiological third stage. Estimated blood loss below 500mls was recorded for 1322 (87.1%) women, with 13 (0.9% of women experiencing a post-partum haemorrhage of greater than 1500mls (seven following spontaneous vaginal birth and nine following operative birth).

Nearly 43% of women had an intact perineum following birth with first- and second-degree perineal tears being most common (25.7 and 27.4% respectively). Severe perineal trauma (3rd and 4th degree tears combined) occurred for 35 (2.3%) women most during spontaneous vaginal birth, including the two 4th degree tears.

Most neonates had an Apgar score greater than or equal to seven at both 1 min and 5 min post-birth (93.7 and 98.8% respectively). Only 37 (2.4% of neonates required admission to NICU following birth. The reasons for NICU admission were recorded as low Apgar scores following shoulder dystocia, non-breathing, grunting, flat baby, respiratory problems, and meconium aspiration. There were no neonatal deaths (Table 1).

### Effect of birthplace at labour and birth on intrapartum events, interventions and maternal and neonatal outcomes

The tertiary maternity unit was the most common planned birthplace for the women in this study at 78%. More than half of nulliparous women (*n* = 704, 59%) chose the tertiary maternity unit for their birthplace over MLU 1 and MLU 2 (*n* = 67, 41% and *n* = 59, 36% respectively), whereas more multiparous women chose MLU 1 and MLU 2 (*n* = 97, 59% and *n* = 106, 64% respectively) over the tertiary maternity unit (*n* = 484, 41%). Midwifery-led units were more likely to adopt water birth than at the tertiary unit: approximately two-thirds of women who used water immersion during labour, both in MLU 1 and MLU 2, had a water birth (*n* = 106, 65% and *n* = 108, 65% respectively) however less than a third of women using water immersion at the tertiary maternity unit had a water birth (*n* = 370, 31%) (Table 2).

**Table 2:** Characteristics and outcomes for women and babies who used water immersion for labour and birth by place of birth

Characteristics and outcomes by place of birth				
	Tertiary maternity unit	Midwife-led unit 1	Midwife-led unit 2	Total
**Caregiver in labour**				
Self -employed Midwife	1077	164	165	1406
Core Midwife	76	0	0	76
Dr. LMC/Core Midwife	35	0	0	35
Total	1188	164	165	1517
**Parity**				
Nulliparous	704	67	59	830
Multiparous	484	97	106	687
Total	1188	164	165	1517
**Gestation at start of labour**				
< 36 weeks	4	0	0	4
36–376 weeks	48	10	14	72
38–396 weeks	502	75	72	649
40–416 weeks	624	79	79	782
> 42 weeks	10	0	0	10
Total	1188	164	165	1517
**Onset of Labour**				
Spontaneous	1095	163	165	1423
Induced	92	1	0	93
Total	1187	164	165	1516
**Membrane Rupture**				
Spontaneous	844	130	154	1128
Artificial	337	32	11	380
Intact	4	1	0	5
Total	1185	163	165	1513
**Pain Relief before entry to water**				
Yes	493	54	37	584
No	695	110	128	933
Total	1188	164	165	1517
**Pain Relief after leaving the pool**				
Yes	503	26	26	555
No	685	138	139	962
Total	1188	164	165	1517
**Length of 1st stage of labour (mins)**				
Mean (SD)	352 (249)	266 (202)	234 (138)	
**Length of 2nd stage of labour (mins)**				
Mean (SD)	62 (66)	22.4 (24)	27 (30)	
**Length of 3rd stage of labour (mins)**				
Mean (SD)	15 (16)	22 (24)	19 (14)	
**Mode of Birth**				
SVD OA	927	164	159	1250
SVD OP	19	0	6	25
Assisted delivery	143	0	0	143
CS	99	0	0	99
Total	1188	164	165	1517
**Birth underwater**				
Yes	370	106	108	584
No	818	58	57	933
Total	1188	164	165	1517
**Position of mother at time of birth**				
Sitting	15	3	7	25
Kneeling	81	17	35	133
Standing	28	5	6	39
Hands and Knees	162	49	28	239
Lateral	21	0	13	34
Semi-reclined	517	73	42	632
Lithotomy	178	4	0	182
Squatting	44	10	29	83
Lying flat	117	1	4	122
Floating	1	0	1	2
Others	2	0	0	2
Total	1166	162	165	1493
**Third stage management**				
Active	775	60	82	917
Physiological	409	103	82	594
Both	4	1	1	6
Total	1188	164	165	1517
**Estimated blood loss**				
0–499	1011	157	154	1322
500–999	131	3	7	141
1000–1499	24	1	1	26
> 1500	11	1	1	13
Total	1177	162	163	1502
**Perineal and/or vaginal wall tear**				
No	492	74	82	648
Yes 1st degree	281	55	54	390
Yes 2nd degree	358	32	25	415
Yes 3rd degree	31	1	1	33
Yes 4th degree	2	0	0	2
Yes Graze	16	2	2	20
Total	1180	164	164	1508
**Episiotomy**				
Yes	206	5	2	213
No	978	157	159	1294
Total	1184	162	161	1507
**Suturing of Tear/Episiotomy**				
Yes	613	56	51	720
No	565	107	112	784
Total	1178	163	163	1504
**Apgar score at 1 min**				
< 7	79	8	3	90
≥ 7	1104	155	162	1421
Total	1183	163	165	1511
**Apgar score at 5 mins**				
> 7	10	1	0	11
≥ 7	1172	162	165	1499
Total	1182	163	165	1510
**Apgar score at 10 mins**				
< 7	0	0	0	0
≥ 7	859	116	67	1042
Total	859	116	67	1042
**Admission to NICU**				
Yes	36	0	1	37
No	1148	163	163	1474
Total	1184	163	164	1511

As expected, all births in the midwife-led units were Spontaneous Vaginal Births (SVB). The gestational age at start of labour was similar across all settings. The use of pain relief during labour differed by place of birth: before entry to the pool/bath 493 (41%) of women in a tertiary maternity unit used pharmacological pain relief, whereas women in the midwife-led units used less (33 and 22% respectively) (Table 2).

In keeping with the strong association between water birth and physiological third stage of labour, 63% (*n* = 103) of women had a physiological third stage of labour at MLU 1 and 50% (*n* = 82) at MLU 2. In the tertiary maternity unit 65% (*n* = 775) of women had an actively managed third stage of labour even.

A second-degree perineal tear was most common across all birth places with 30% at the tertiary unit, and 20 and 15% at the two midwife-led units respectively. Episiotomy was rarely used at the midwife-led units. Women in the tertiary maternity unit, where the water birth rate was much lower than MLU 1 and MLU 2, showed higher numbers of episiotomy and suturing than women in the MLUs (Table 2).

The Apgar scores at 1, 5 and 10 min across all the birth places were most usually ≥ seven. Most neonates born to women who used water immersion for labour and birth did not require admission to NICU (98%).

## Discussion

This study described the maternal characteristics, intrapartum events, interventions, and maternal and neonatal outcomes of women who used water immersion during labour and birth at one New Zealand DHB during the period 2009–2014. The data revealed 84% of women who used water immersion for labour and birth across all three birth settings in this DHB had a spontaneous vaginal birth (Nulliparous 74.3%, Multiparous 95.7%). This finding is important when compared with the DHB statistics reported in their 2009 annual clinical report that showed 41.3% (1643/3975) of all women who went to term, had a spontaneous labour then had a spontaneous vaginal birth (9). Of note, in this same report, only 34.5% (630/1826) of all primiparous women who went to term, had a spontaneous labour went on to have a spontaneous vaginal birth. The New Zealand national average for spontaneous vaginal birth was reported to be 65.2% in 2014 [9].

The New Zealand Clinical Indicators report outcomes for the ‘standard primipara’ across all DHBs and facilities [10]. They define the standard primipara as: women aged 20–34 years old at the time of giving birth who are giving birth for the first time (parity = 0) at term (37–41 weeks’ gestation) where the outcome of the birth is a singleton baby, the presentation is cephalic and there have been no recorded obstetric complications that are indications for specific obstetric interventions (p. 8). This definition is used in data analysis to identify a group of women who are ‘low risk’, and for whom interventions and outcomes should be similar across all birthing facilities and regions. The New Zealand Clinical Indicators report a decrease in the proportion of standard primiparae who had a spontaneous vaginal birth, and continued variation between regions during the period 2009–2014, the same period as the data collected in this study.

The rate of water immersion for labour and birth was very small overall. Despite the availability of a bath or birth pool in every birthing room at the tertiary unit, approximately 7.7% of women used this during labour and birth. The finding that more women have a water birth in the MLUs than at the tertiary unit is likely to be influenced by the philosophy of LMC led community-based care. A midwifery-led continuity of care model in New Zealand means midwives can support women to birth at home, primary birthing units or in the hospital [11]. Most women birthing at the tertiary maternity unit in this study also had a community-based LMC (many of whom also practice at the MLUs) or core midwife providing their intrapartum care. Despite this, the rates of water immersion for labour and birth in the tertiary maternity unit are less than half the rates in the MLU. An explanation for the lower rate of water immersion for labour and birth in the tertiary maternity unit warrants review of the environment and culture of the tertiary maternity unit, which is geared more towards the needs of the institution and influenced by a biomedical model of care, impacts on decision-making for women and midwives around timeframes, interventions and pain relief options, particularly the availability of epidural analgesia [1]. It is critical that these differences are discussed with women antenatally [1]. Support for healthy physiological birthing within the tertiary maternity should be a focus of education for both midwives and doctors [1] including the practice of supporting women to use water immersion for labour and birth.

The 2009 systematic review (Cluett and Burns, 2009) concluded that labouring in water reduces the need for pharmacological pain relief [11]. This study also found a reduction in the use of pain relief in the form of inhalation/Entonox, epidural, and opioid in the group of women who used water immersion for labour and birth. It is interesting to explore whether water immersion in and of itself influences a reduction in pain and the need for pain relief. An earlier qualitative study indicated that it was not necessary to give birth in the water to achieve the benefits of water immersion such as relaxation, increased ability to cope with pain, reduced fear and the to maintain control over the birth process [12].

In the literature, outcomes related to perineal trauma are mixed. Nutter and colleagues (2014) report that there is a decreased likelihood of severe perineal trauma (3rd and 4th-degree tears) associated with water birth [13]. In this study, there were only 35 (2%) third, and fourth-degree tears which compares favourably with the number of third and fourth-degree tears reported at the DHB during 2015 (4.2%) [14]. The rate of intact perineum was 43% which is significantly higher than the 17.9% rate reported for this DHB in 2015 [9].

Blood loss estimation is mainly subjective in most instances. The mean estimated blood loss over 1000mls low is low for all birth settings. While there were no other additional measures used in this study to determine the impact of blood loss such as haemoglobin estimation [15, 16], this finding is consistent with findings of Nutter and colleagues, 2014 [13].

As would be expected for this group of low-risk women, neonatal complications were few, with only 37 (2%) of babies admitted to the NICU. The low rate of neonatal admission is in keeping with the findings of the 2016 systematic review of neonatal outcomes following water birth [17].

### Limitations

The results presented are from a sample of women who gave birth in one DHB in New Zealand, and as such, the findings are only indicative of the practice in these settings. The results might not represent all women who used water immersion for labour and birth at the DHB during the time frame of this study. Anecdotally, during the study period, we were aware that not all midwives completed documentation all the time, but were unable to establish the extent of this, nor to enforce it. No data for maternal age or ethnicity were collected. A further limitation is the lack of a control group of low-risk women who met the eligibility criteria but did not use the bath or birth pool during labour. To reduce the proportion of missing data, a review of the PIMS and the clinical records occurred.

### Implications for practice

The differences in outcomes by place of birth should prompt a discussion around what else is happening to impact the decisions of women and midwives. Focused education on water immersion for labour and birth for all midwives and doctors is warranted as well as antenatal education that provides evidence for the impact of place of birth and caregiver on outcomes for women and babies.

### Implications for research

More research is required to explore factors impacting on women’s decision-making around the use of water immersion for labour and birth as well as women’s satisfaction. It would be beneficial to explore the factors influencing midwives to offer water immersion for labour and birth, and the barriers and facilitators to offering this service in the tertiary maternity environment.

## Conclusion

Water immersion for labour and birth is a positive intervention that benefits well women with uncomplicated pregnancies. This study shows that water immersion for labour and birth in a midwife-led unit with a community-based lead maternity care midwife results in excellent outcomes for women and infants. Water immersion for labour and birth also provides an essential option for women who have a desire to have a spontaneous vaginal birth. The positive outcomes generated from water birthing indicate that this simple intervention may be a useful solution to address the high intervention rates in New Zealand’s birthing population in tertiary and community settings. Midwives in both tertiary and community-based midwives should, therefore, campaign for improved accessibility to water immersion and water birth for women birthing in all birthing setting.

## Supplementary information

**Additional file 1: Supplementary File 1.** Water immersion outcome data collection sheet.

## Data Availability

The datasets used and/or analysed during the current study are available from the corresponding author on reasonable request.

## Abbreviations

ARM: Artificial rupture of the membranes
BP: Blood pressure
C/S: Caesarean section
CTG: Cardiotocograph
DHB: District Health Board
HDEC: Health and Disability Ethics Committee
IOL: Induction of labour
IT: Information technology
LMC: Lead Maternity Carer
MLU: Midwifery led unit
MMPO: Midwifery and Maternity Provider Organisation
NICU: Neonatal Intensive Care Unit
OP: Occipito-anterior position
OP: Occipito-posterior position
PIMS: Patient information system
PPH: Postpartum haemorrhage
SD: Standard deviation
SFD: Small for dates
SROM: Spontaneous rupture of membranes
SVB: Spontaneous vaginal birth
TENS: Transcutaneous electrical nerve stimulator
