# Supplementary figures and images for: Getting into the water: a prospective observational study of water immersion for labour and birth at a New Zealand District Health Board
Source: BMC Pregnancy Childbirth. 2020 May 20;20:312. doi: 10.1186/s12884-020-03007-6 (PMC7238728; doi:10.1186/s12884-020-03007-6)

Supplementary File One: Water immersion outcome data collection sheet


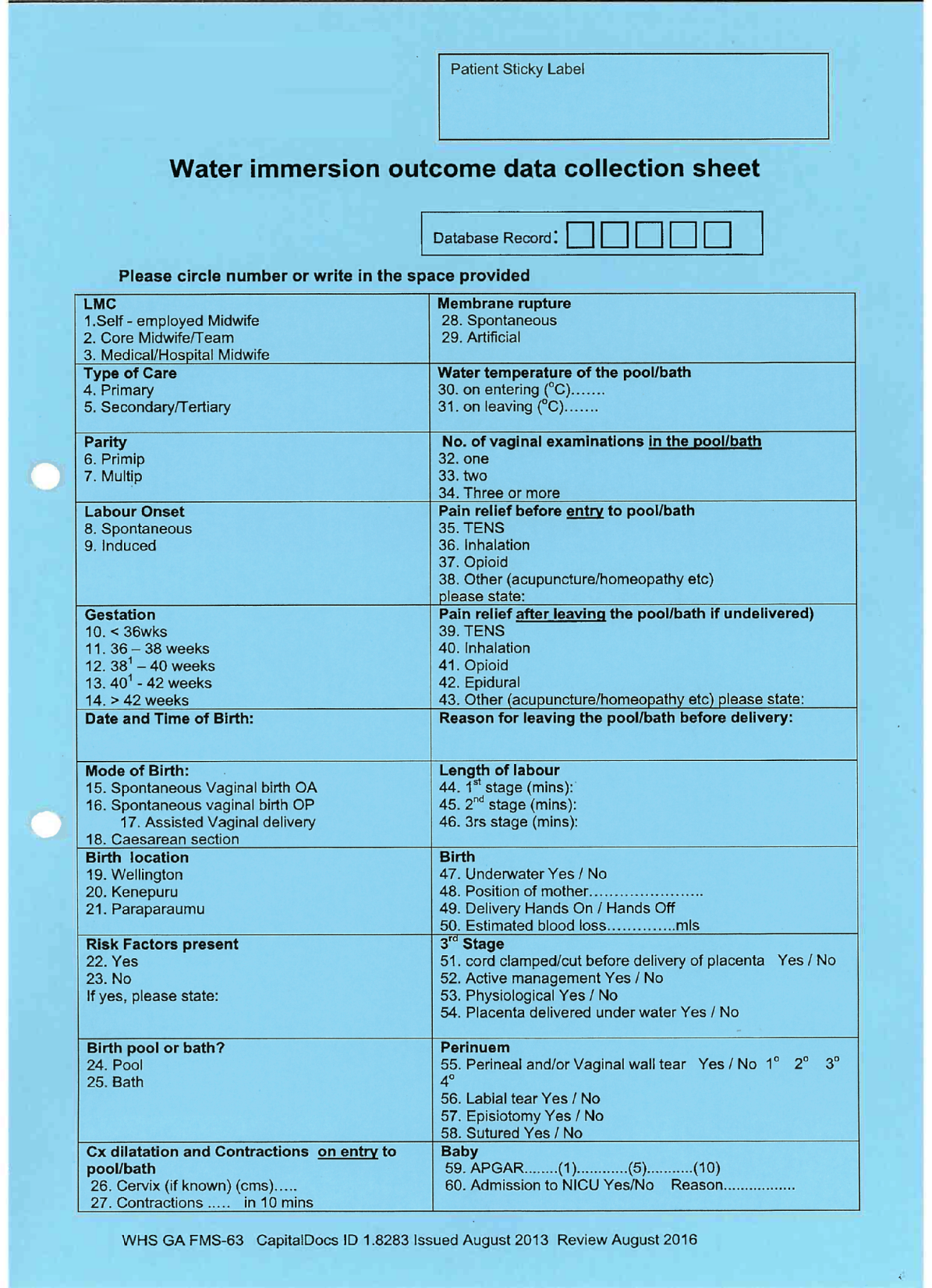

Supplement: Supplementary file 1 — Additional file 1: Supplementary File 1. Water immersion outcome data collection sheet. [file 12884_2020_3007_MOESM1_ESM.docx]
